# A Novel Fractional-Order Chaotic Phase Synchronization Model for Visual Selection and Shifting

**DOI:** 10.3390/e20040251

**Published:** 2018-04-04

**Authors:** Xiaoran Lin, Shangbo Zhou, Hongbin Tang, Ying Qi, Xianzhong Xie

**Affiliations:** 1 College of Computer Science, Chongqing University, Chongqing 400044, China; 2 Chongqing/MII Key Lab. of Computer Network and Communication Technology, Chongqing 400044, China; 3 College of Mathematics and Information Engineering, Chongqing University of Education, Chongqing 400065, China

**Keywords:** cognitive informatics, fractional-order, chaotic phase synchronization, active control

## Abstract

Visual information processing is one of the fields of cognitive informatics. In this paper, a two-layer fractional-order chaotic network, which can simulate the mechanism of visual selection and shifting, is established. Unlike other object selection models, the proposed model introduces control units to select object. The first chaotic network layer of the model is used to implement image segmentation. A control layer is added as the second layer, consisting of a central neuron, which controls object selection and shifting. To implement visual selection and shifting, a strategy is proposed that can achieve different subnets corresponding to the objects in the first layer synchronizing with the central neuron at different time. The central unit acting as the central nervous system synchronizes with different subnets (hybrid systems), implementing the mechanism of visual selection and shifting in the human system. The proposed model corresponds better with the human visual system than the typical model of visual information encoding and transmission and provides new possibilities for further analysis of the mechanisms of the human cognitive system. The reasonability of the proposed model is verified by experiments using artificial and natural images.

## 1. Introduction

Cognitive informatics is an interdisciplinary research area of information sciences and cognitive sciences, which studies the internal information processing mechanisms and processes of natural intelligence in human brains and minds [1]. Visual information processing in the brain is the foundation of cognitive informatics [2]. The human visual system can select salient objects from a complex background and shift attention between different significant objects. Visual selection and shifting is a basic activity of the human visual system. The activity includes two stages: bottom-up and top-down. The first stage mainly depends on the unconscious behavior of retinal cells [3] and the second stage relies on control by the visual cortex. In these neural activities, synchronization plays an important role. Many physiological experiments have confirmed that synchronization occurs in the brain activity of humans, monkeys, mice and cats [4,5,6,7,8]. Synchronization is an important mechanism in image segmentation and feature binding. In the synchronization system, objects are represented by temporal correlation and spatially distributed neurons. Temporal correlation is encoded by the synchronization of oscillators that represent some features of an object and is termed “oscillatory correlation” [9]. Oscillatory correlation has been used to implement image segmentation and feature binding [10,11,12]. The oscillatory correlation mechanism is used to select objects and segment images in a network in which the oscillators oscillate in a limited cycle [13,14]. In other literature, chaotic synchronization is used to bind and segment images using complete and phase synchronization [15,16,17]. In real experiments, complete synchronization is rarely observed and the conditions required to achieve complete synchronization are strict. Phase synchronization is a basic mechanism for visual feature binding, as confirmed by multiple studies [18,19]. Therefore, we will use phase synchronization to construct a novel visual attention model.

There are two types of computer visual attention models. With the first type, location-based models, attention is only focused on a point or a small area and only one neuron is activated. With the second type, object-based models, visual attention can shift from one object to another. This type of model is supported by many behavioral and neurophysiological experiments [9,20,21]. The existing models discussed above are integer-order models and do not include any of the control units. The cognitive system, including the control unit, is more similar to the human cognitive system. The simulation system provides some help in the analysis of the human cognitive system. Some unique properties can be better represented by fractional-order models than by integer-order models [22] because a fractional-order model can exhibit more complex dynamic phenomena than an integer-order model [23]. The fractional time-delay systems exhibit superiority in their description of identification and the memory system [24,25]. Thus, in this paper, we depict visual attention using a fractional-order model with a control unit.

Phase synchronization in chaotic systems is that the phase difference of two systems is kept bounded over time while their amplitudes may be completely uncorrelated [26]. Phase synchronization in a chaotic system and network has been widely investigated [27,28,29]. Most of the literature focuses on phase synchronization among two or more coupled chaotic systems but a chaotic network synchronized with a single chaotic oscillator is still rarely reported. For synchronization among hybrid systems [30,31], we will propose a new synchronization strategy in which the chaotic network is considered a hybrid system composed of the linear superposition of phase synchronization oscillators. The hybrid system will achieve synchronization with the central neuron by active control.

For visual perceptions, we will propose a novel two-layer model. The first layer of the model, scene segmenting, is based on a model proposed by Zhao [17]. In Zhao et al.’s model, visual shift is based on the coupling strength changing over time. The control effect of a central neuron is not involved. In another study [32], the visual attention model is also two-layer, implementing visual selection and shift based on the resonance frequency. In our model, the second layer consists of a central unit, with which it implements visual selection and shift by chaotic control. The neuron in the second-layer can simulate the cerebral cortex neuron to control the first layer represented by the different objects by time division phase synchronization. The two-layer model designed in this way will be closer to the human visual system. This paper contributes three findings: Firstly, a synchronization strategy between a network and a single chaotic system is proposed; Secondly, chaotic control theory and fractional calculus are introduced into the model of visual attention; and finally, a fractional-order visual attention model with a control unit is established.

The remainder of this paper is organized as follows: in Section 2, the fractional-order chaotic system is presented and time division phase synchronization in the two-layer model is implemented; in Section 3, the visual attention model is described; the simulation results are shown in Section 4 and our conclusions are presented in Section 5.

## 2. Phase Synchronization of Fractional-Order Chaotic Systems

The role of synchronization in brain information processing has received support from neurobiologists [5]. The processing of oscillator synchronization is the foundation of temporal coding, called oscillatory correlation. The feature detectors are represented by oscillators and binding of the oscillators is represented by an assembly by synchrony. The oscillators representing different assemblies are desynchronous. Complete chaotic synchronization has been used to code in image segmentation. Phase synchronization has a less-restrictive condition than complete synchronization and is easier to implement. Thus, we use phase synchronization to realize the function of temporal coding.

Phase synchronization is when the phase difference of two systems is kept bounded while their amplitudes are uncorrelated. The unwrapped phase ψ is depicted as

(1) $$\psi =\Upsilon \left(arctan\left(\frac{y}{x}\right)\right)$$

where *x* and *y* are the variables of the chaotic system; Υ is the unwrap operation and ψ is an increasing variable. For convenience, we also use phase to represent an unwrapped phase.

### 2.1. Fractional-Order Chaotic Network

**Figure 1:**
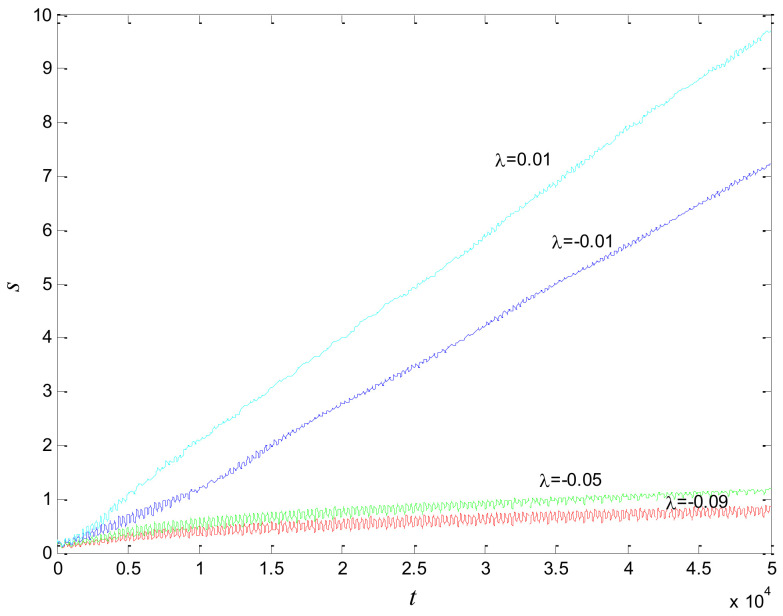
Phase standard deviation with different coupling strengths.

A network coupled by fractional-order Rössler oscillators can achieve phase synchronization [27]. The coupled system can be modeled as

(2) $$\{\begin{array}{c} D^{\alpha }x_{j}=-o_{j}y_{j}-z_{j}+\lambda \left(2x_{j}-x_{j-1}-x_{j+1}\right)\\ D^{\alpha }y_{j}={ο}_{j}x_{j}+ay_{j}\\ D^{\alpha }z_{j}=b+z_{j}\left(x_{j}-c\right) \end{array}$$

where *λ* and *o_j_* are the coupling strength and the system frequency, respectively; α = 0.9 is the fractional order of every oscillator and *a* = 0.48, *b* = 0.6, *c* = 6 are the system parameters. The state of synchronization is affected by the coupling strength *λ*. The phase standard deviations for different coupling strengths are shown as in Figure 1. As the coupling strength increases in absolute value, the phases of the coupled oscillators tend towards consistency. The phase standard deviation of the system is minimized at *λ* = −0.09.

### 2.2. Phase Synchronization between the Central System and the Chaotic Network

Time division synchronization is a means of communication that has been widely used [33,34]. Here, we use this method to control the central system and achieve phase synchronization with different subnets (groups) in the networks at different times. The response system (central system or central unit) is constructed as

(3) $$\{\begin{array}{c} D^{\alpha }x_{r}=-o_{r}y_{r}-z_{r}+u_{xr}\\ D^{\alpha }y_{r}=o_{r}x_{r}+ay_{r}+u_{yr}\\ D^{\alpha }z_{r}=b+z_{r}\left(x_{r}-c\right)+u_{zr} \end{array}$$

The driven system is a network composed of many oscillators (Rössler oscillators) as follows:

(4) $$\{\begin{array}{c} D^{\alpha }x_{j}=-o_{j}y_{j}-z_{j}+\lambda \left(2x_{j}-x_{j-1}-x_{j+1}\right)\\ D^{\alpha }y_{j}=o_{j}x_{j}+ay_{j}\\ D^{\alpha }z_{j}=b+z_{j}\left(x_{j}-c\right) \end{array}$$

The network is composed of two groups of oscillators whose frequencies are at different intervals. The frequencies of the two groups are within [1.01, 1.02] and [0.98, 0.99], respectively. The oscillators are synchronized with other neurons in the same group by the appropriate coupling strength. The central oscillator synchronizes firstly with the oscillators whose phase velocities are faster and then with the second group. The active control method described in Reference [29] is used to implement phase synchronization. If the central oscillator implements synchronization with every oscillator in the two groups, the control function *u*_(*x*, *y*, *z*)_ will be extremely complicated. Liu et al. [30,31] have realized hybrid synchronization between heterogeneous chaotic systems. Inspired by this idea, we construct a hybrid system composed of a group of oscillators and carry out phase synchronization between the central system and the hybrid system. The constructed hybrid system is the mean of system variables of the oscillators in the network, depicted by Formula (5):

(5) $$\{\begin{array}{c} w_{x}=\frac{x_{k}+x_{k+1}\ldots x_{k+n}}{n}\\ w_{y}=\frac{y_{k}+y_{k+1}\ldots x_{k+n}}{n}\\ w_{z}=\frac{z_{k}+z_{k+1}\ldots z_{k+n}}{n} \end{array}$$

The error system is depicted as

(6) $$\{\begin{array}{c} e_{x}=x_{r}-w_{x}\\ e_{y}=y_{r}-w_{y}\\ e_{z}=z_{r}-w_{z} \end{array}$$

Phase synchronization between the response system and the hybrid driven system is implemented by active control. Derivation of the control function *u*_(*x*, *y*, *z*)_ can be seen presented in Appendix A.

**Figure 2:**
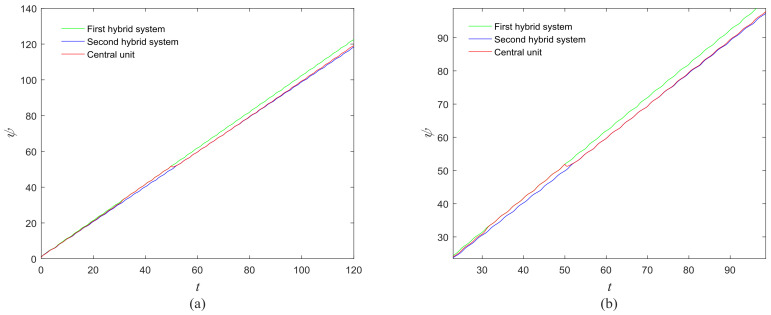
Phase growth of the proposed fractional-order systems. (**a**) The central unit and two hybrid systems; (**b**) Detail view of phase jump in the visual shift.

The phase growth of the hybrid system is the same as the oscillators in the group, as shown in Figure 2. The hybrid system can represent the states of the oscillators in a group. This means that phase synchronization between a chaotic oscillator and a chaotic network can be translated to phase synchronization between a chaotic oscillator and a hybrid system. The response system is set up to synchronize with different groups of neurons at different times. When *t* = *t*_1_, the first hybrid system is controlled by the central unit to realize phase synchronization. Suppose the two systems maintain synchrony for *t_s_*. When *t* = *t*_1_ + *t_s_* + 0.01, the response system starts to synchronize with the second hybrid system. The phase time-division synchronization is depicted in Figure 2.

**Figure 3:**
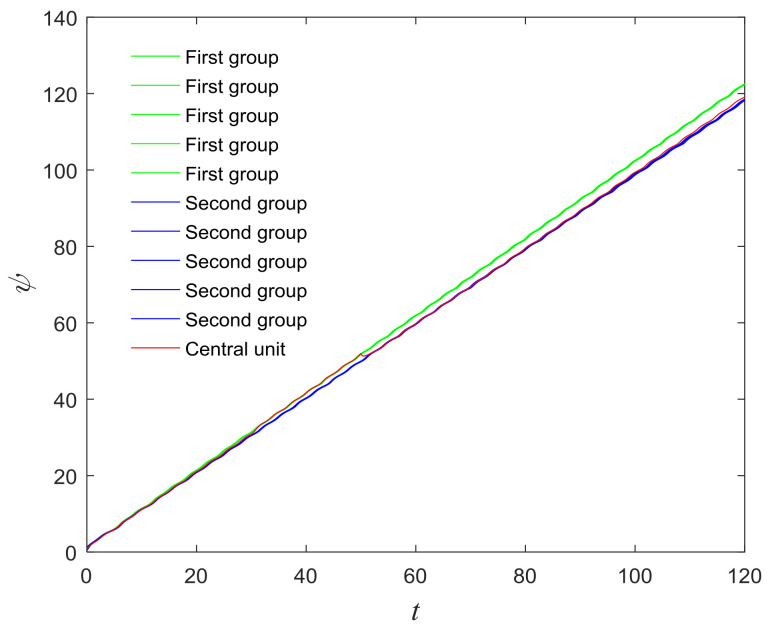
Phase growth of the central unit and randomly selected oscillators from each group.

**Figure 4:**
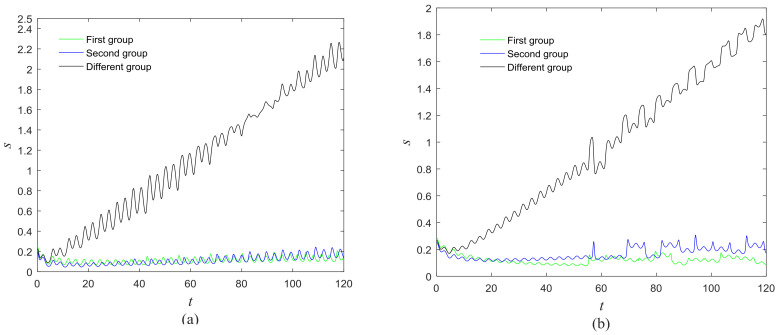
Phase standard deviation of groups over time. (**a**) Our proposed model; (**b**) The segmentation model in [17].

**Figure 5:**
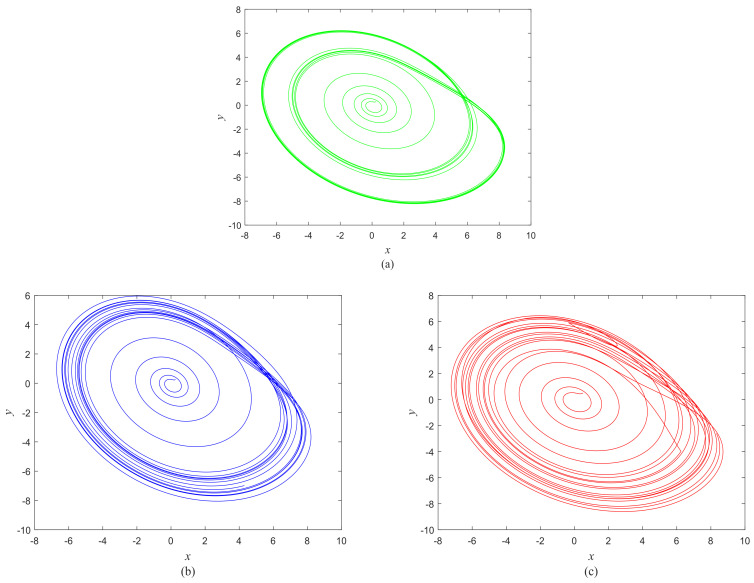
Attractors of different systems. (**a**) The second hybrid system; (**b**) The first hybrid system; (**c**) The control system.

The phase growth of the central unit with two hybrid systems is shown in Figure 2a. The phase growth of the first hybrid system and the central system are the same from *t* = 30 to *t* = 50. This indicates that the two systems are in phase synchronization in this time span. The red line in Figure 2a stands for the phase of the central unit. The phase jumps at *t* = 30 and *t* = 50, as shown in Figure 2b. This means the phase of the central unit has a transition. After *t* = 80, the control unit does not control the second group of neurons and the phase growth of the central unit differs from the second group of neurons. Figure 3 shows that the central unit achieves phase synchronization with randomly selected oscillators in each group. The phase standard deviations of oscillators representing the same object are small, as shown in Figure 4. This also indicates that the oscillators representing the same object are synchronized. Figure 5 depicts the attractor of each of the three systems. Figure 4a,b shows that the phase standard deviations representing the same group obtained by our model changes are smaller than that obtained by the model proposed in [17]. It shows that the consistency of the oscillators in the same group is better. Comparing Figure 5a,b, it can be seen that the phase of the first hybrid system changes more quickly than the second one does and the central unit has an obvious transition. Thus, we have implemented phase synchronization between the single central unit and the network composed of coupled oscillators.

## 3. Model Description 

**Figure 6:**
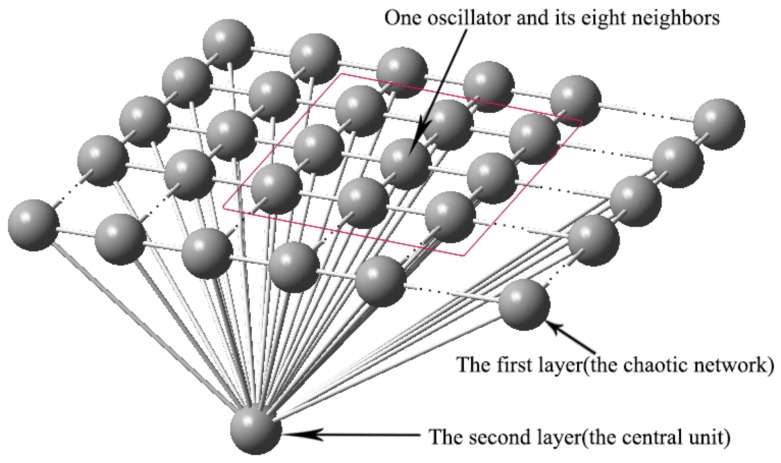
Proposed two-layer model.

In this section, the visual shift model is described by graph and text. The proposed model consists of two-layer Rössler oscillators and is shown in Figure 6. In the first layer, every oscillator couples with eight neighbors, except oscillators on the boundary. The mathematical model of the network is described by Equation (7). The main function of the first layer is to represent the formation of visual information. The structure of this layer is the same as the oscillator network in [17] and the way of connecting the network can encode topology and can separate different objects in the image. The number of oscillators is selected according to the size of the image. The second layer, composed of a central neuron oscillator, acts as the central neuron. The central neuron is coupled with different groups of neurons in the first layer at different times. The different groups of neurons represent different objects. The central neuron synchronizes with different groups of neurons by active control methods and realizes the communication with the imaging neurons. The model is described by Equations (7) and (8):

The first layer:

(7) $$\{\begin{array}{c} D^{\alpha }x_{j,k}=-o_{j,k}y_{j,k}-z_{j,k}+\lambda_{j,k}^{+}\sigma^{+}x_{j,k}+\lambda_{j,k}^{-}\sigma^{-}x_{j,k}\\ D^{\alpha }y_{j,k}=o_{j,k}x_{j,k}+ay_{j,k}\\ D^{\alpha }z_{j,k}=b+z_{j,k}\left(x_{j,k}-c\right) \end{array}$$

The second layer:

(8) $$\{\begin{array}{c} D^{\alpha }x_{r}=-o_{r}y_{r}-z_{r}+u_{xr}\\ D^{\alpha }y_{r}={ο}_{r}x_{r}+ay_{r}+u_{yr}\\ D^{\alpha }z_{r}=b+z_{r}\left(x_{r}-c\right)+u_{zr} \end{array}$$

where (*j*, *k*) stands for the oscillator in the *j*-th row and *k*-th column of the two-dimensional topology, *j* ∈ [1, *M*], *k* ∈ [1, *N*]; *M*, *N* is the size of the input image; $\lambda_{j,k}^{+}$ and $\lambda_{j,k}^{-}$ are the positive and negative coupling strength; and $u_{xr},\;u_{yr},\;u_{zr}$ are the active control functions of the second layer (response system). The details of the control functions can be seen in Appendix A. The positive and negative coupling terms are $\sigma^{+}x_{j,k}$ and $\sigma^{-}x_{j,k}$, with

$$\begin{array}{ll} \sigma^{\pm }x_{j,k}= & \Delta_{j-1,k-1;j,k}(x_{j-1,k-1}-x_{j,k})+\Delta_{j-1,k;j,k}(x_{j-1,k}-x_{j,k})\\ & \;+\Delta_{j-1,k+1;j,k}+\Delta_{j,k-1;j,k}(x_{j,k-1}-x_{j,k})\\ & \;+(x_{j-1,k+1}-x_{j,k})\Delta_{j,k+1;j,k}(x_{j,k+1}-x_{j,k})\\ & \;+\Delta_{j+1,k-1;j,k}(x_{j+1,k-1}-x_{j,k})+\Delta_{j,k-1;j,k}(x_{j,k-1}-x_{j,k})\\ & \;+\Delta_{j,k+1;j,k}(x_{j,k+1}-x_{j,k}) \end{array}$$

where $\Delta_{j,k;p,q}=\{\begin{array}{c} 1,\;\mathrm{if}\;\mathrm{oscillator}\;\left(j,k\right)\;\mathrm{is}\;\mathrm{coupled}\;\mathrm{to}\;\left(p,q\right)\\ 0,\;\mathrm{otherwise} \end{array}$, $o_{j,k}$ is used to code the contrast of the pixel (*j*, *k*), because the contrast sensitivity is one of the information processing mechanisms in the human visual system. $o_{j,k}$ is determined by Δo, which depends on the image and *C*_*j*,*k*_, as follows:

(9) $$o_{j,k}=1-0.5*\Delta o+\Delta o*C_{j,k}$$

*C_j,k_* is the weighted average of the absolute difference:

(10) $$C_{j,k}=\frac{\sum_{l}w^{l}\left|f_{j,k}^{l}-f_{avg}^{l}\right|}{\sum_{l}w^{l}}$$

where $f_{j,k}^{l},f_{avg}^{l}$ are the values of pixel(*j*, *k*) and the average value for feature *l*, respectively. In this paper, feature refers to the gray intensity and the three *R*, *G*, *B* color channels. The four weights $w^{l}$ for the four features are set to be 0.5, 1/6, 1/6, 1/6. The positive and negative coupling strengths are described by Equations (11) and (12):

(11) $$\lambda_{j,k}^{+}=\lambda_{max}^{+}\times exp\left(-\frac{{\left(1-C_{j,k}\right)}^{2}}{2\sigma^{2}}\right),$$

(12) $$\lambda_{j,k}^{-}=\lambda_{max}^{-}\times \left(1-exp\left(-\frac{{\left(1-C_{j,k}\right)}^{2}}{2\sigma^{2}}\right)\right),$$

where $\lambda_{max}^{+}$ and $\lambda_{max}^{-}$ are the max positive and negative coupling strength. σ is set according to the image. In the second layer, $o_{r}$ is the mean value of all $o_{j,k}$ in the first layer. Other parameters are the same as for the first layer.

**Figure 7:**
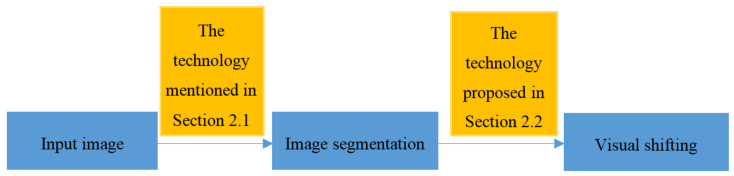
The block diagram of the proposed model.

The block diagram of the proposed model is shown as in Figure 7. The blue blocks are the functional modules and the yellow blocks are the technical measures to implement the next functional modules.

## 4. Experiments

In this section, simulation results obtained by the proposed model are demonstrated. Artificial and natural images are used as the experimental example to evaluate the performance of the model. In the experiment, the max positive and negative coupling strengths are 0.05 and 0.02, respectively. In the experiments with natural images, a Gaussian filter is used for image denoising.

**Figure 8:**
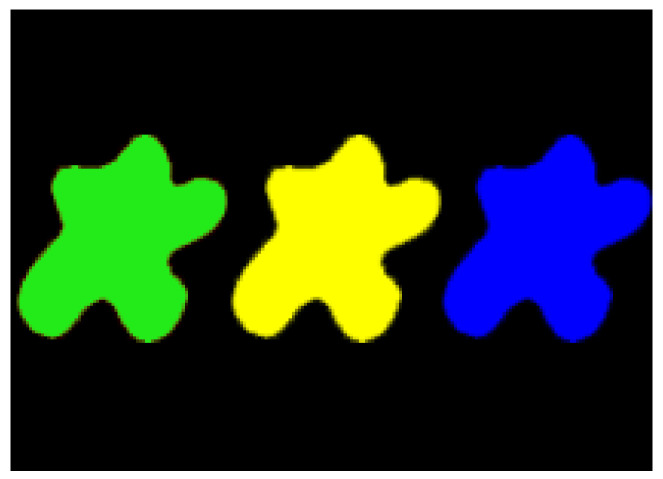
Artificial image with three objects.

**Figure 9:**
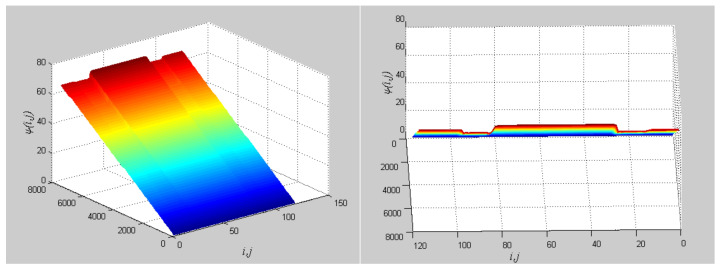
Different perspectives for phase diagrams. The left describes the entire phase change, and the right describes the final phase profile.

**Figure 10:**
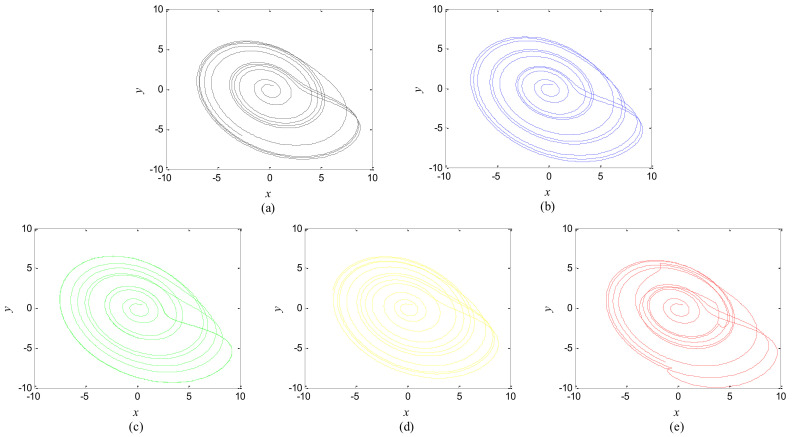
Attractors corresponding to the objects in Figure 8 and the central unit. (**a**) Background; (**b**) Blue object; (**c**) Green object; (**d**) Yellow object; (**e**) Central unit.

**Figure 11:**
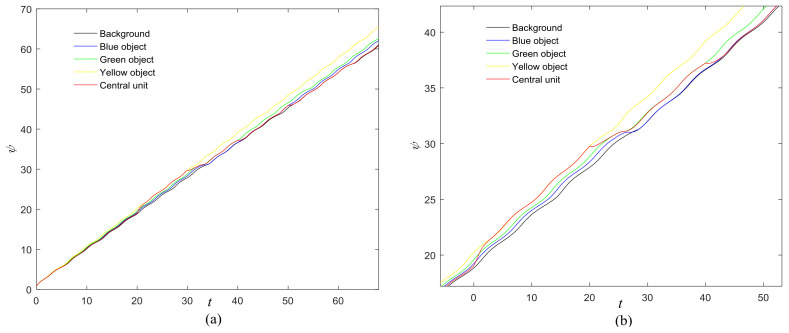
(**a**) Phase growth corresponding to the objects in Figure 8; (**b**) Detail view of phase jump in the visual shift.

**Figure 12:**
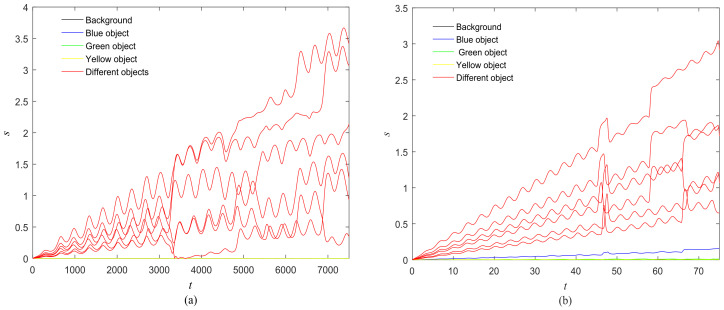
Phase standard deviation of objects in Figure 8 over time. (**a**) Our proposed model; (**b**) The segmentation model in [17].

The first artificial image has three objects, as shown in Figure 8. To improve computing speed, the image is resized to 210 × 151 pixels. The oscillators representing the same object achieve phase synchronization due to the coupling strength and homologous frequency. On the other hand, the neurons representing different objects are desynchronized. Figure 9 shows the differences in phase among oscillators representing different objects from different perspectives. In the (*i*, *j*) axis, a random selection of oscillators is shown corresponding to the blue object, background, yellow object, the background and the green object one after the other from right to left, respectively. The oscillators representing yellow objects have the fastest phase growth, closely followed by the oscillators representing green objects, then oscillators representing blue objects and finally oscillators representing the background. The attractors of the hybrid systems corresponding to different objects are shown in Figure 10. The moving trajectories of oscillators for different objects are distinctive. In the experiment, the central unit and the first layer oscillators begin to oscillate at the same time. When the central unit finds the most salient object whose phase velocity is the fastest, it will couple with these oscillators and achieve synchronization with them by the active control method. The duration of synchronization is attention span, denoted by *F_t_* (in this experiment *F_t_* = 10). When the fixation time is over, attention will shift to the second salient object. The shift strategies are the same until the attention scans all objects in the scene. The attention shift process is shown as in Figure 11. When *t* = 20, the central neuron will start phase synchronization with the oscillators corresponding to the yellow object and will then maintain synchronization with them for *F_t_*. At *t* = 30.01, the central unit will couple with the neurons representing the green object. During the entire process, the phase of the central neuron transitions four times. The control unit’s attractor is shown as in Figure 9e and the transition is obvious. The phase growth between different objects is distinctive. The phase standard deviation of each object is shown in Figure 12. The standard deviation is low between the same objects and high between different objects. In Figure 9, Figure 10 and Figure 11, it can be seen that for more salient objects, the phase growth is faster. This result of this experiment is consistent with the conclusion in [17]. It suggests that the fractional-order network layer can be used for image segmentation.

We compare our model with the model proposed in [17]. If the phases of oscillators representing same object are similar and the phases of oscillators representing different objects are very different, the segmentation results may be satisfactory. In this paper, we do three comparison tests to compare the segmentation results, as shown in Figure 12. In Figure 12, the phase standard deviations representing the same object obtained by our model changes less than that obtained by the model proposed in [17] and the phase standard deviations representing different objects obtained by our model grow more rapidly than that obtained by the model proposed in [17]. We also calculate the entropy of the segmentation results of Figure 8, which represents the probability of a particular information occurrence. If the entropy is smaller, the consistency of the segmentation results is better. Using our model and Zhao’s model, the segmentation results showed the local entropy to be 5.8986 and 6.4393, respectively. In general, for segmentation, the performance of our proposed model is better than that of the model in [17].

**Figure 13:**
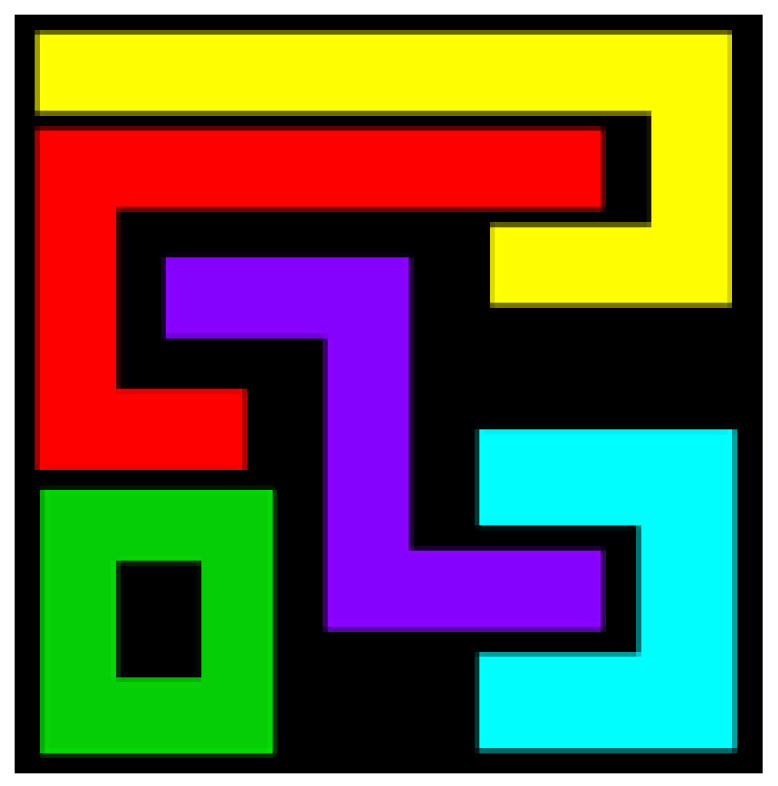
Artificial image with five objects.

**Figure 14:**
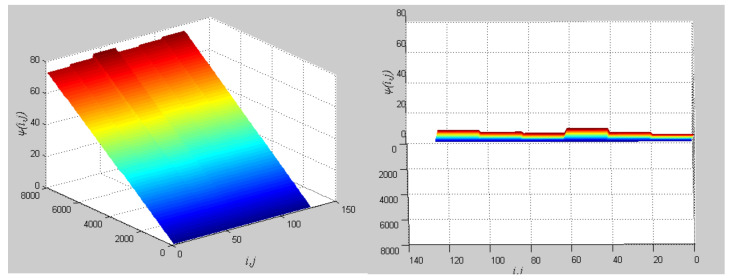
Different perspectives for phase diagrams. The left describes the entire phase change, and the right describes the final phase profile.

**Figure 15:**
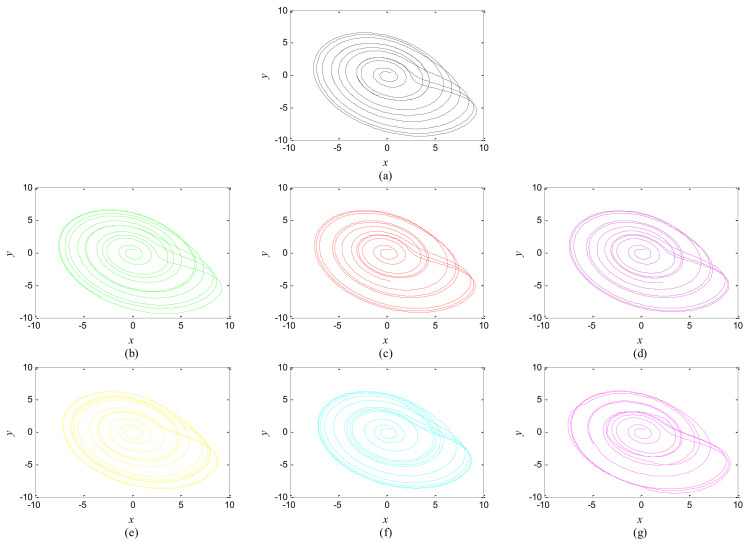
Attractors corresponding to the objects in Figure 13 and the central unit. (**a**) Background; (**b**) Green object; (**c**) Red object; (**d**) Purple object; (**e**) Yellow object; (**f**) Azure object; (**g**) Central unit.

**Figure 16:**
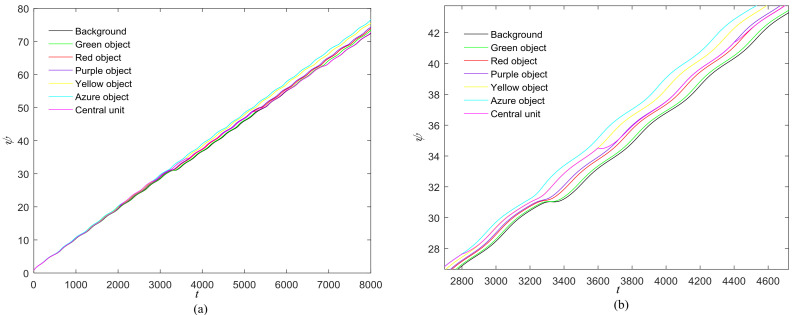
(**a**) Phase growth corresponding to the objects in Figure 13; (**b**) Detail view of phase jump in the visual shift.

**Figure 17:**
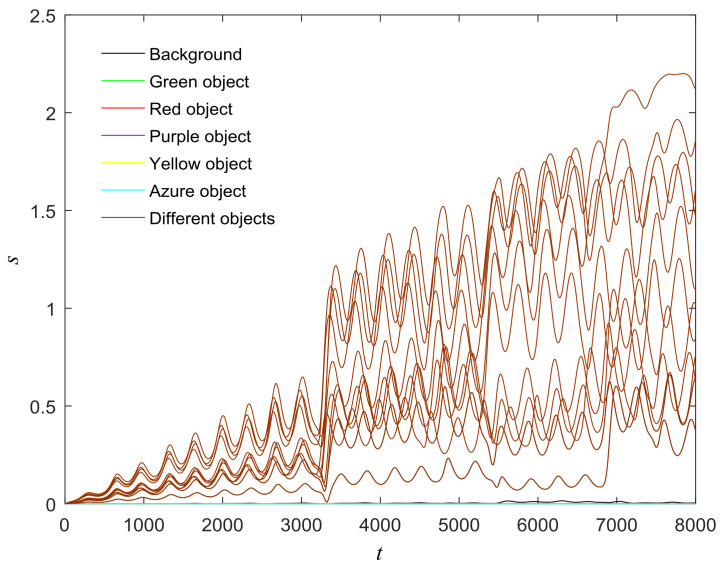
Phase standard deviation for objects for Figure 13.

The proposed model can also be used in scenes with more objects, such as those shown in Figure 13, Figure 14, Figure 15, Figure 16 and Figure 17. There are six different phase types in Figure 14: yellow object, purple object, green object, azure object, red object and background. The attractors corresponding to different-colored objects are different. The central unit, represented by the pink line, transitions six times. The central unit’s attractor also reflects the phase transitions. The standard deviation is low between the same objects and high between different objects. The results confirm that the proposed model is reasonable.

**Figure 18:**
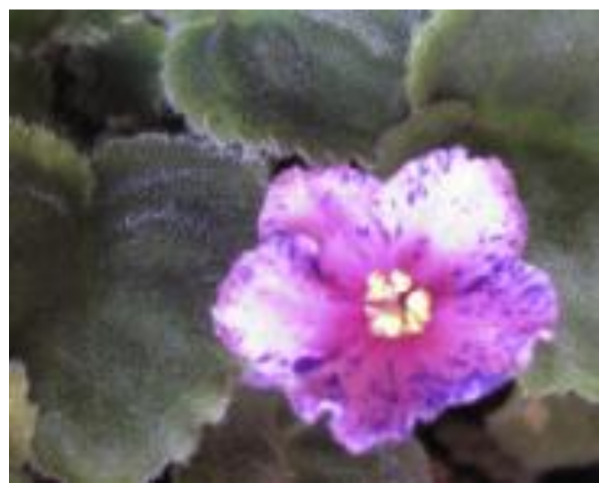
Natural image: “flower”.

**Figure 19:**
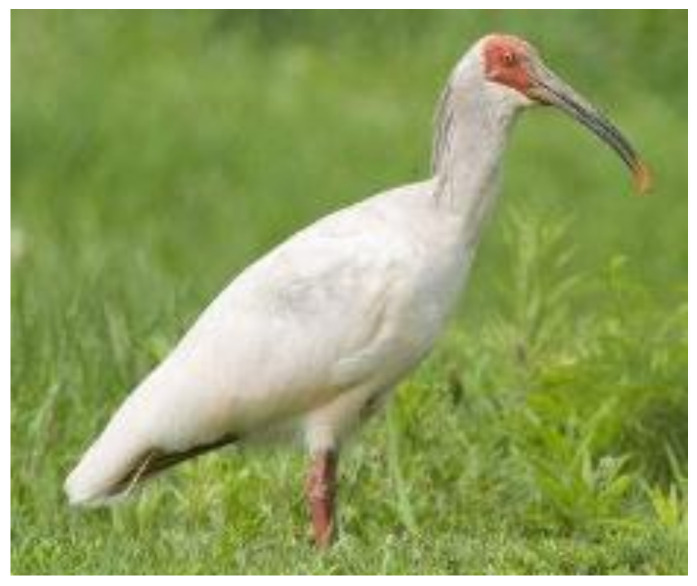
Natural image: “bird”.

**Figure 20:**
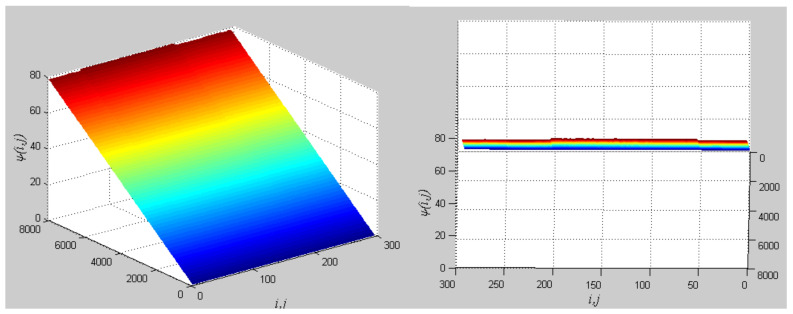
Different perspectives for phase diagrams. The left describes the entire phase change, and the right describes the final phase profile.

**Figure 21:**
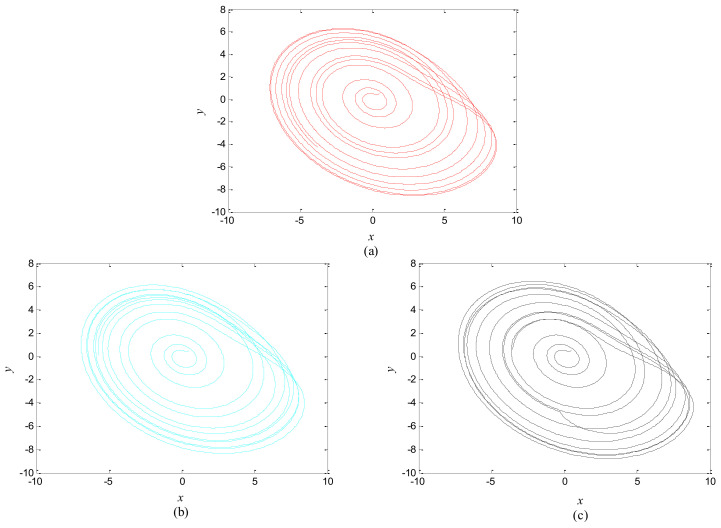
Attractors corresponding to the objects in Figure 18 and the central unit. (**a**) Flower; (**b**) Leaves; (**c**) Central unit.

**Figure 22:**
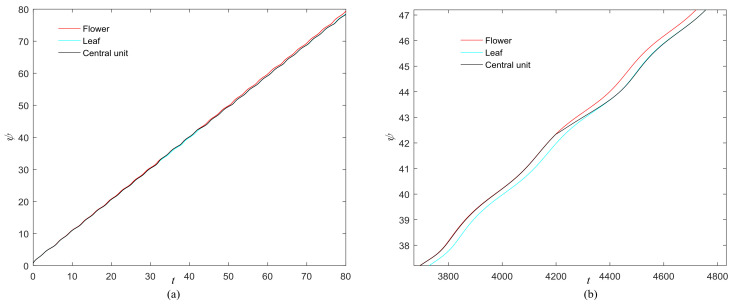
(**a**) Phase growth corresponding to the objects in Figure 18; (**b**) Detail view of phase jump in the visual shift.

**Figure 23:**
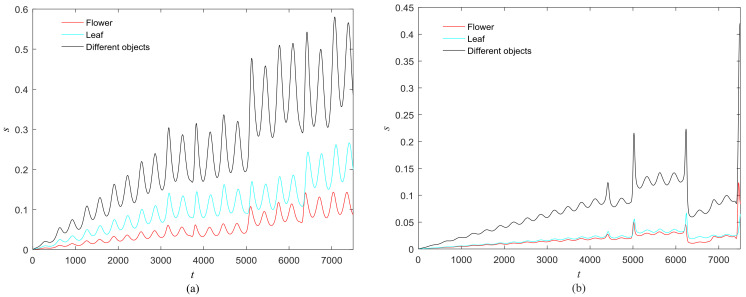
Phase standard deviation for objects in Figure 18 over time. (**a**) Our proposed model; (**b**) The segmentation model in [17].

**Figure 24:**
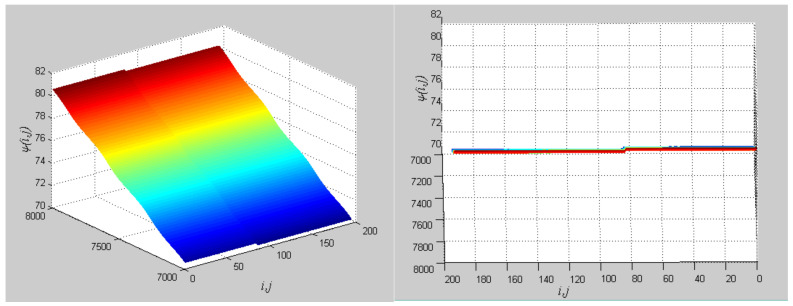
Different perspectives for phase diagrams. The left describes the entire phase change, and the right describes the final phase profile.

**Figure 25:**
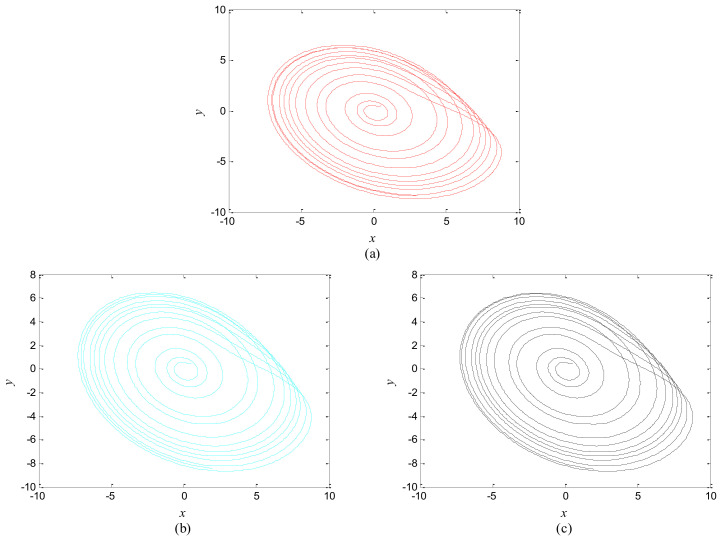
Attractors corresponding to the objects in Figure 19 and the central unit. (**a**) Bird; (**b**) Grass; (**c**) Central unit.

**Figure 26:**
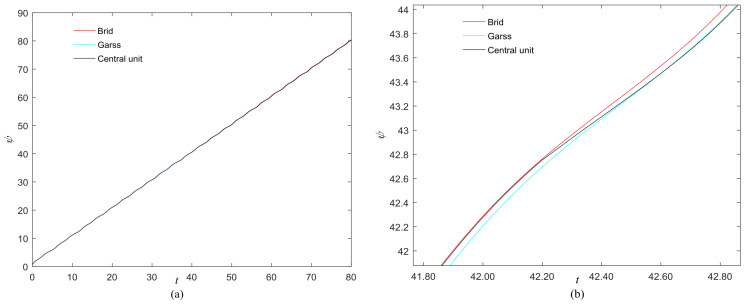
(**a**) Phase growth corresponding to the objects in Figure 19; (**b**) Detail view of phase jump in the visual shift.

**Figure 27:**
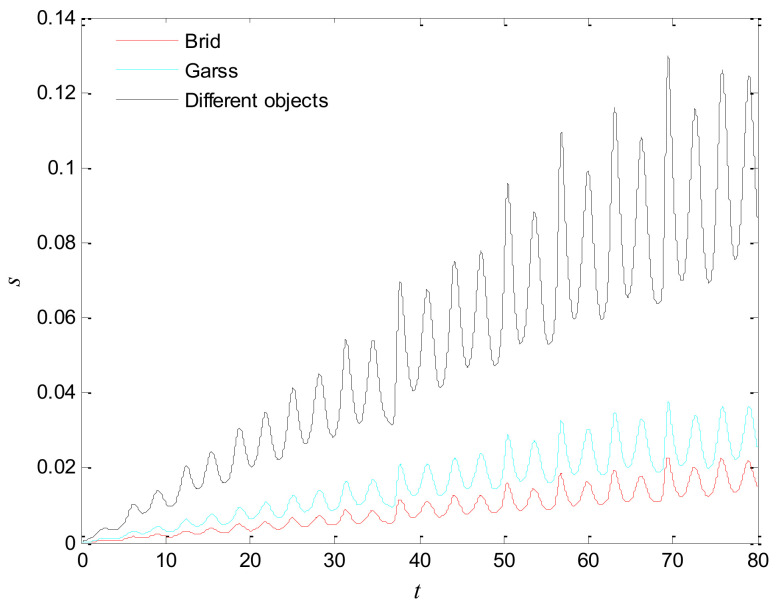
Phase standard deviation for objects in Figure 19.

Furthermore, we employ the natural images to test the proposed model. The natural images are shown in Figure 18 and Figure 19 and the experimental results are shown in Figure 20, Figure 21, Figure 22, Figure 23, Figure 24, Figure 25, Figure 26 and Figure 27. It can be seen that the phase growth is distinct for different objects. The control unit is also synchronized with the “attention object” during the control time. More detailed views are included as Figure 22b and Figure 26b, in which the phase transition is obvious. Note in Figure 23 and Figure 27 that the phase standard deviation between different objects is relatively small. This is because the contrasts between pixels derived from Equation (9) are small. The small contrasts lead to small phase differences between oscillators representing different objects. However, the central neuron can still synchronize with different oscillators at different times. The phase standard deviations representing the different objects obtained by our model grows more rapidly than that obtained by the model proposed in [17], as shown in Figure 23b Thus, the proposed model is also effective for visual selection and shifting in natural images.

**Figure 28:**
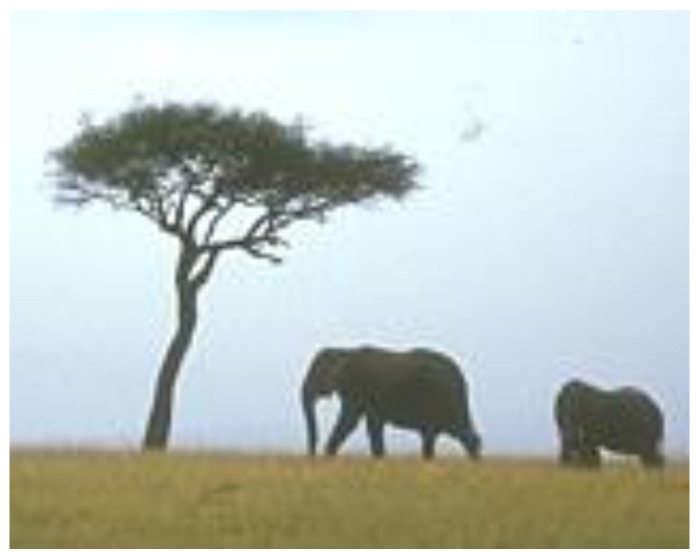
“Rhinoceros” form BSDS500 dataset.

**Figure 29:**
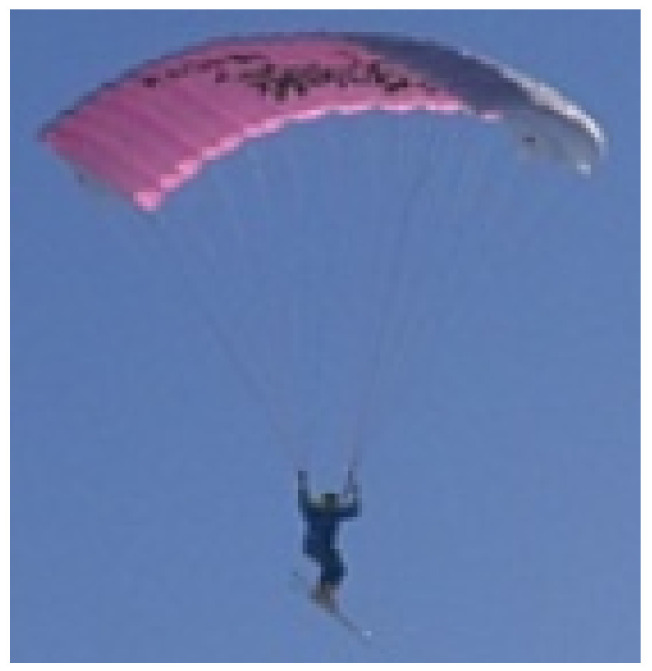
“Parachuting” form BSDS500 dataset.

**Figure 30:**
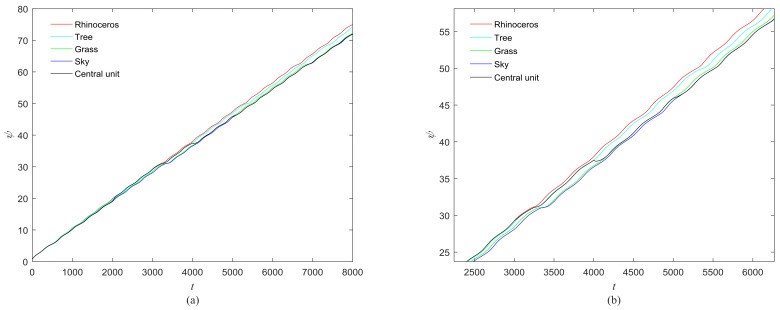
(**a**) Phase growth corresponding to the objects in Figure 28; (**b**) Detail view of phase jump in the visual shift.

**Figure 31:**
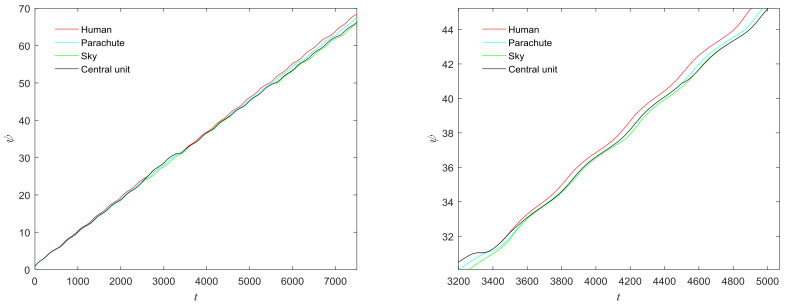
(**a**) Phase growth corresponding to the objects in Figure 29; (**b**) Detail view of phase jump in the visual shift.

We also selected the images from the BSDS500 (Berkley Segmentation Data Set) [35] to test the performance of the proposed model. The results for Figure 28 and Figure 29 are shown in Figure 30 and Figure 31. The central unit has 4, 3 times transitioning to synchronize with the “attention object” during the control time in Figure 30 and Figure 31. We also calculate the mean entropy of the segmented object. For Figure 28, the mean entropies obtained by our model and the model in [17] are 4.3606 and 4.39965, respectively. For Figure 29, the mean entropies obtained by the mentioned models are 5.1878 and 5.2262, respectively. For the two images, the mean entropy of segmented objects obtained by our model are small. This means that the performance of our proposed model is better than that of the model in [17] for image segmentation.

## 5. Conclusions

In this paper, a two-layer model is presented for attention shift. The first layer of the model is used for image segmentation. The second layer, acting as a control function of cognitive systems, implements attention shift by time division phase synchronization between the network and an oscillator. In this processing, a strategy is designed for time division phase synchronization between the network and an oscillator. The proposed model consists of visual and central nervous system cells. The introduction of a control function can make the proposed model closer to the human visual system than previous models and the fractional-order oscillators can better portray the mechanism of visual shift than the model with integer order ones.

The main current work presented in this paper is a novel model for visual selection and shifting. In future research, we will try to introduce a competition mechanism to the model to make the attention shift more consistent with visual search theory [36,37].

## Appendix A

To determine the control functions *u*, we subtract (4) from (3) and obtain

(A1) $$\{\begin{array}{c} D^{\alpha }e_{x}=-o_{r}y_{r}-z_{r}-\frac{\left(-o_{j}y_{j}-z_{j}+\Gamma_{j}\right)\ldots +(-o_{n}y_{n}-z_{n}+\Gamma_{n})}{n}+u_{x}\\ D^{\alpha }e_{y}=o_{r}x_{r}-ay_{r}-\frac{\left(o_{j}x_{j}-ay_{j}\right)+\ldots \left(o_{n}x_{n}-ay_{n}\right)}{n}+u_{y}\\ D^{\alpha }e_{z}=z_{r}x_{r}-cz_{r}-\frac{\left(z_{j}x_{j}-cz_{j})+\ldots (z_{n}x_{n}-cz_{n}\right)}{n}+u_{z} \end{array},$$

where $\Gamma_{j}=\lambda \left(2x_{j}-x_{j-1}-x_{j+1}\right)$. Every neuron is phase synchronized with the central unit. The Formula (7) can be rewritten as

(A2) $$\{\begin{array}{c} D^{\alpha }e_{x}=\left((-o_{r}y_{r}-z_{r}-\left(-o_{j}y_{j}-z_{j}+\Gamma_{j}\right)\right)+\ldots (-o_{r}y_{r}-z_{r}-(-o_{n}y_{n}-z_{n}+\Gamma_{n})))/n+u_{x}\\ D^{\alpha }e_{y}=\left((o_{r}x_{r}-ay_{r}-\left(o_{j}x_{j}-ay_{j}\right)\right)+\ldots (o_{r}x_{r}-ay_{r}-\left(o_{n}x_{n}-ay_{n}\right)))/n+u_{y}\\ D^{\alpha }e_{z}=\left((z_{r}x_{r}-cz_{r}-(z_{j}x_{j}-cz_{j}\right))+\ldots (z_{r}x_{r}-cz_{r}-(z_{n}x_{n}-cz_{n})))/n+u_{z} \end{array}$$

(A3) $$\{\begin{array}{c} u_{x}=\left((o_{r}y_{j}-o_{j}y_{j}+\Gamma_{j}\right)\ldots +(o_{r}y_{n}-o_{n}y_{n}+\Gamma_{n}))/n+V_{x}\\ u_{y}=\frac{\left(o_{r}x_{j}-o_{j}x_{j}\right)\ldots +\;\left(o_{r}x_{n}-o_{n}x_{n}\right)}{n}+V_{y}\\ u_{z}=-z_{r}x_{r}+\frac{z_{j}x_{j}\ldots +z_{n}x_{n}}{n}+V_{z} \end{array},$$

the Formula (8) becomes

(A4) $$\{\begin{array}{c} D^{\alpha }e_{x}=-o_{r}e_{y}-e_{z}+V_{x}\\ D^{\alpha }e_{y}=o_{r}e_{x}-ae_{y}+V_{y}\\ D^{\alpha }e_{z}=-ce_{z}+V_{z} \end{array}.$$

The linear functions can be written as

(A5) $$\{\begin{array}{c} V_{x}=o_{r}e_{y}+e_{z}\;+\theta_{x}e_{x}\\ V_{y}=-o_{r}e_{x}+ae_{y}\;+\theta_{y}e_{y}\\ V_{z}=\;ce_{z}+\theta_{z}e_{z} \end{array},$$

where $\theta_{x},\;\theta_{y},\;\theta_{z}$ are the eigenvalues of the system (A4). To satisfy the condition $\left|\arg\left(\theta_{x,y,z}\right)\right|>\alpha \pi /2$, $\theta_{x},\;\theta_{y}\;$ and $\theta_{z}$ are set to −1, −1, −1, respectively. Then, the error system (A4) is stable and the two systems can synchronize.

This completes the derivation of the control function.
